# Campbell biology (edited by Lisa Urry, Michael Cain, Steven Wasserman, Peter Minorsky and Jane Reece)

**DOI:** 10.1186/s40709-020-00127-0

**Published:** 2020-12-09

**Authors:** Gangxu Shen

**Affiliations:** 1 School of Chinese Medicine for Post-Baccalaureate, I-Shou University, Kaohsiung, Taiwan; 2 National Changhua University of Education, Changhua, Taiwan

**Keywords:** Campbell biology, University students, Postgraduate medical examinations

## Abstract

*Campbell Biology* is divided into eight units and 56 chapters. The organization and size of this book are appropriate and easy for first-year university students and help them to learn and digest the content. *Campbell Biology* is currently among the best biology books and it is listed with the best shelling textbooks. *Campbell Biology* is mainly for first-year university students, but it is also an important book for postgraduate medical examinations. Moreover, some high school students may use it as an essential reference book. In its current edition, the latest information in various fields has been added, such as the basal body, which was previously called the 9*3 type microtube arrangement but now has been renamed as the 9 + 0 type in Chapter 6. The updates in molecular biology are closer to the current situation, such as the addition of information on next-generation sequencing and CRISPR/Cas9 in Chapter 20. This content can enable readers to acquire the latest knowledge. Reading this book and understanding the information presented in its pages is very helpful for the future life science professionals. Thus, *Campbell Biology* is very valuable textbook in the field of biology.

## Book details

Campbell Biology, 11th Lisa Urry, Michael Cain, Steven Wasserman, Peter Minorsky, Jane Reece.

Pearson Education, 2017

ISBN13: 978-0-134-09341-3

Biology is a compulsory course in a university’s biomedicine-related departments. Biology includes cytology, energetics, genetics, molecular biology, botany, evolution, ecology, and taxonomy. Biology is necessary to prepare for detailed study in various fields.

## The readership of Campbell Biology

*Campbell Biology* is mainly for first-year university students, but it is also an important book for postgraduate medical examinations. Moreover, some high school students may use it as an essential reference book. Although the content may be difficult for high school students, it is suitable for first-year university students. However, the content may be too basic for candidates appearing for post-baccalaureate Chinese medicine and Western medicine examinations. Because of the fierce competition in these examinations, books with much more advanced content are often preferred. Sometimes, the entrance exam questions for the post-baccalaureate medicine department are taken from more professional books, such as those closely related to biochemistry, molecular biology, genetics, or ecology.

## What is new in *Campbell Biology*

In this edition of *Campbell Biology*, the latest information in various fields has been added, such as the basal body, which was previously called the 9*3 type microtube arrangement but now has been renamed as the 9 + 0 type in Chapter 6 [1]. The updates in molecular biology are closer to the current situation, such as the addition of information on next-generation sequencing and CRISPR (clustered regularly interspaced short palindromic repeat)/Cas9 in Chapter 20 [1]. This content can enable readers to acquire the latest knowledge.

## The organization of Campbell Biology

*Campbell Biology* is divided into eight units and 56 chapters [1]. The organization and size of this book are appropriate and easy for first-year university students and help them to accept and learn the content. *Campbell Biology* is currently among the best biology books and it is listed with the best shelling textbooks*.* Of course, some content lack depth compared with more specialized books, but this is understandable given its main target is first-year university students. For more in-depth content, readers may refer to other books, such as *Gene* 12th, *Molecular Cell Biology* (Lodish et al.), *Lehninger Principles of Biochemistry* 7th, *Immunobiology* (Janeway et al.), *Principles of Genetics* (Snustad and Simmons), *Vander’s Human Physiology* 15th, *Elements of Ecology* (Smith and Smith), and *Brock Biology of Microorganisms* 15th.

## Suggestions on Campbell biology content

### Unit 2 The cell

Chapter 6, page 102: The fact that ribosomes are not membrane bounded means that they cannot technically be considered organelles [1]; however, a number of books, such as *Starr Biology* [2], describe ribosomes as organelles. I therefore suggest that the book make readers aware that even among experts, opinions differ.

Chapter 7, page 138: The term secondary active transport, which is commonly used in other textbooks on physiology [3] and biochemistry [4] is referred to in *Campbell Biology* as cotransport [1]. To prevent confusion, I suggest using the common term, secondary active transport.

### Unit 3 Genetics

Chapter 15, page 310: On a few genes, methylation has been shown to activate the expression of the allele. One example is the insulin-like growth factor 2 (Igf 2) gene, on which the methylation of particular cytosines on the paternal chromosome leads to the expression of the paternal Igf 2 allele [1]. Note however that in *Gene 12th* [5], the description is quite different: “The ICR is methylated on the paternal allele, where Igf 2 is active. The ICR is unmethylated on the maternal allele, where Igf 2 is inactive.” I suggest that readers be informed that methylation occurs on the ICR, rather than on Igf 2. This will help to avoid confusion when they read the descriptions in other texts, such as *Brooker Biology*: “Igf 2 is methylated on the maternal allele, where Igf 2 is inactive” [6].

Chapter 20, pages 429–430: Embryonic stem cells (ES cells) are pluripotent, which means that they are capable of differentiating into many different cell types [1]. Note however, that Figure 20.20 ascribes to ES cells the ability to generate “all embryonic cell types” [1]. I recommended modifying the text as “many different cell types” to ensure consistency between the main text and figure caption.

### Unit 5 The evolutionary history of biological diversity

Chapter 27, pages 578: The process of bacterial conjugation by which DNA is transferred has yet to be fully elucidated. In fact, recent evidence suggests that DNA passes directly through the hollow pilus. In *Principles of genetics* [7], it is clearly stated that F pili are involved in establishing cell contact, rather than the transfer of DNA. I therefore recommended that *Campbell Biology* make it clear that the sex pilus is involved only in cell contact and not in the transmission of DNA.

### Unit 6 Plant form and function

Chapter 35, page 778: The ABC model of flower formation involves formation of the four types of floral organs [1]. The functions of MADS-box gene have been extensively studied in *Arabidopsis thaliana*, where ABCDE genes specify the fate of floral organs by the combinatorial ABCDE model [8, 9]. In this model, A, B and C proteins interact with E proteins [10]: A and E produces sepals; A, B and E produces petals; B, C and E produces stamens; C and E produces carpels [10–13]. Therefore, I suggest that *Campbell biology* should change the content of ABC model to ABCDE model.

### Unit 7 Animal form and function

Chapter 46, page 1032: Animal embryo development process is zygote, cleavage, and blastocyst [1]. But before the formation of blastocyst, there is a period called Morula [3, 14, 15]. This period of Morula should not be omitted.

## Why should read this book

The fact that *Campbell Biology* outlines the foundations of life science makes it a must-read for all life science professionals. If you ever expect to apply for a post-baccalaureate position as in a medical department, *Campbell Biology* should be on your list of essential reading.

## Data Availability

Campbell biology.

## Abbreviations

CRISPR: Clustered regularly interspaced short palindromic repeat.
Igf 2: Insulin-like growth factor 2
ES cells: Embryonic stem cells
